# Factors associated with adherence to dietary prescription among adult patients with chronic kidney disease on hemodialysis in national referral hospitals in Kenya: a mixed-methods survey

**DOI:** 10.1186/s41100-019-0237-4

**Published:** 2019-09-11

**Authors:** Rose Okoyo Opiyo, Peter Suwirakwenda Nyasulu, Joyce Olenja, Moleen Zunza, Kim A. Nguyen, Zipporah Bukania, Esther Nabakwe, Alexander Mbogo, Anthony Omolo Were

**Affiliations:** 1 School of Public Health, College of Health Sciences, University of Nairobi, Nairobi, Kenya; 2 East African Kidney Institute, College of Health Sciences, University of Nairobi, Nairobi, Kenya; 3 Division of Epidemiology and Biostatistics, Department of Global Health, Faculty of Medicine and Health Sciences, Stellenbosch University, Cape Town, South Africa; 4 Centre for Public Health Research – KEMRI, Nairobi, Kenya; 5 Department of Child Health and Paediatrics, Moi University, Eldoret, Kenya; 6 Kenyatta National Hospital, Nairobi, Kenya; 7 Department of Internal Medicine, College of Health Sciences, University of Nairobi, Nairobi, Kenya

**Keywords:** Adherence, Diet, Nutrition, Renal, Hemodialysis, Kenya, Mixed-methods study, Food

## Abstract

**Introduction:**

Adherence to dietary prescriptions among patients with chronic kidney disease is known to prevent deterioration of kidney functions and slow down the risk for morbidity and mortality. This study determined factors associated with adherence to dietary prescription among adult patients with chronic kidney disease on hemodialysis.

**Methods:**

A mixed-methods study, using parallel mixed design, was conducted at the renal clinics and dialysis units at the national teaching and referral hospitals in Kenya from September 2018 to January 2019. The study followed a *QUAN* + *qual* paradigm, with quantitative survey as the primary method. Adult patients with chronic kidney disease on hemodialysis without kidney transplant were purposively sampled for the quantitative survey. A sub-sample of adult patients and their caregivers were purposively sampled for the qualitative survey. Numeric data were collected using a structured, self-reported questionnaire using Open Data Kit “Collect software” while qualitative data were collected using in-depth interview guides and voice recording. Analysis on STATA software for quantitative and NVIV0 12 for qualitative data was conducted. The dependent variable, “adherence to diet prescription” was analyzed as a binary variable. *P* values < 0.1 and < 0.05 were considered as statistically significant in univariate and multivariate logistic regression models respectively. Qualitative data were thematically analyzed.

**Results:**

Only 36.3% of the study population adhered to their dietary prescriptions. Factors that were independently associated with adherence to diet prescriptions were “flexibility in the diets” (AOR 2.65, 95% CI 1.11–6.30, *P* 0.028), “difficulties in following diet recommendations” (AOR 0.24, 95% CI 0.13–0.46, *P* < 001), and “adherence to limiting fluid intake” (AOR 9.74, 95% CI 4.90–19.38, *P* < 0.001).

**Conclusions:**

For patients with chronic kidney disease on hemodialysis, diet prescriptions with less restrictions and requiring minimal extra efforts and resources are more likely to be adhered to than the restrictive ones. Patients who adhere to their fluid intake restrictions easily follow their diet prescriptions. Prescribed diets should be based on the individual patient’s usual dietary habits and assessed levels of challenges in using such diets. Additionally, diet adherence messages should be integrated with fluid limitation messages. Further research on understanding patients’ adherence to fluid restriction is also suggested.

## Background

Chronic kidney disease (CKD) is a global public health problem and is now on the rise gradually [1]. About 850 million people are affected worldwide. This represents 11 to 13% of the total global population [2–4]. In Sub--Saharan Africa, currently, about 16% of the population is affected [5], a rise from 14% reported in 2014 [6]. The prevalence of CKD in Eastern Africa is also high, currently reported at 14% [7]. Kidney disease is now ranked as the sixth fastest growing cause of mortality globally, with over 2.4 million deaths per year [8].

In CKD, the kidney functions progressively decline, leading to a decrease in glomerular filtration rate, slowdown in removal of waste from the bloodstream, and accumulation of waste in the blood as well as changes in requirements and utilization of various nutrients [9]. Dietary adaptations for key nutrients, particularly carbohydrates, proteins, sodium, potassium, phosphorus, and fluid intake, are necessary to reduce the risk for morbidity and mortality in patients with CKD [10]. For patients on hemodialysis, limiting the intake of certain foods is important in order to reduce the accumulation of these metabolic wastes in the blood and to reduce the development of comorbidities such as hypertension, proteinuria, and other health complications of the heart and bones [11]. The dietary restrictions are recommended to prevent deterioration of kidney functions and thus slowing down the risk for morbidity and mortality [10]. However, most CKD patients encounter difficulties in adjusting to the recommended diet for their disease condition. Importantly, more than 50% of the dialysis patients consumed inadequate dietary intake for most nutrients [12, 13] on one hand and excess intake of phosphorus, sodium, calcium, and potassium on the other [12]. Evidence on dietary restrictions shows that adherence is a challenge for many patients with CKD [10, 14–16], with more than half of adult patients with CKD not adhering to their dietary prescriptions.

The World Health Organization defines adherence as the extent to which a person’s behavior in taking medication, following a diet, and/or executing lifestyle changes corresponds with agreed recommendations for their disease condition [17]. Clark-Cutaia and colleagues in the USA [18] observed that young females had more difficulties adhering to their hemodialysis dietary regimens. In Australia, dietary change among CKD patients involves several factors including cooking skills, ability to read and comprehend food labels, and cost and availability of fresh food [14]. Adherence appears to be a multidimensional phenomenon where patient-related, condition-related, socio-economic, therapy-related, and health care-related factors [17, 19] all exert their forces on the CKD patient, contributing to non-adherence to both dietary and medication guidelines. Yet, often, it is the patient who is blamed for non-adherence. The patient-related factors, health perceptions, and psychosocial factors have also been associated with no adherence to diet and fluid recommendations among patients with end-stage renal disease in Jordan [20]. Chironda and Bhengu [21] also observed that non-adherence to dietary prescription among CKD patients in South Africa was due to inability to afford the prescribed diet and unwillingness to avoid some of the recommended foods.

Most accessible studies reporting on adherence to dietary restrictions among patients with CKD are outside the African continent [7, 22–24]. In Kenya, documented research on adherence to dietary prescriptions among adults with CKD on hemodialysis is either non-existent or, if there is, non-accessible. Yet, currently, over 10,000 cases are diagnosed annually with CKD in Kenya, and it is estimated that 4.8 million Kenyans will be suffering from kidney disease by 2030 [25]. According to the Kenya Renal Association, the number of patients with kidney disease undergoing chronic hemodialysis increased from 300 in the year 2006 to 2400 in 2018 [26] in Kenya. Since nutrition is the most modifiable lifestyle factor in the management of CKD, it is important that adherence to dietary prescription and the food environment factors that affect accessibility, availability, acquisition, and preparation of food in the Kenyan context are well understood in order to prescribe the most appropriate modified diet for these patients. The Kenyan food environment may not be similar to what is found in other parts of the world; hence, the proposed solutions to non-adherence problem from existing studies may not be applicable in Kenya. The aim of this study was therefore to determine factors associated with adherence to dietary prescription among adult patients with chronic kidney disease on hemodialysis in national referral and teaching hospitals in Kenya. This will guide intervention strategies during nutrition counseling.

## Methods

### Study design and setting

This was a mixed-methods study, using convergent parallel design where quantitative and qualitative data were collected concurrently [27]. The advantage of combining quantitative and qualitative methods to answer a single research problem is to gain a more complete perspective and to best understand the problem and thus pave ways for appropriate strategies for addressing the problem [28]. Accordingly, the present study followed a *QUAN* + *qual* paradigm with quantitative approach as the primary method [27, 28], and the data were integrated so as to provide a comprehensive understanding around the factors influence the adherence to dietary recommendations/prescriptions in CKD patients on hemodialysis.

The study was conducted at the renal clinics and dialysis units within Kenyatta National Hospital (KNH) and Moi Teaching and Referral Hospital (MTRH). These are the two main public teaching and referral hospitals in Kenya with an average renal patients’ attendance of 100 per month in KNH and 70 per month in MTRH. The KNH is located in Nairobi, the capital and largest city of Kenya, while MTRH is located in Eldoret Town, Uasin Gishu County, Kenya. These two referral hospitals were selected as the study sites due to cultural and ethnic diversity that define food preferences of patients attending the renal clinics in Kenya.

### Quantitative approach

A facility-based cross-sectional survey was conducted from September 2018 to January 2019. We used a structured, self-reported questionnaire designed using the Open Data Kit (ODK) “Collect software” which was uploaded and administered on smartphones. The data collection program had a built in quality control mechanism. The inclusion criteria consisted of “adult patients aged 18 years, with CKD, on hemodialysis, with stable health condition, and able to communicate well at the time of data collection.” Patients who provided voluntary informed consent were included. Patients with a history of kidney transplant, in unstable condition, and with no caregiver were excluded.

The sample size for quantitative study was 331. We used OpenEpi software [29] to calculate the sample size which was based on an assumption of 50% adherence to dietary recommendations among adults with CKD on hemodialysis due to patient-related factors [17, 30]. We assumed an odds ratio of 0.5 and a statistical power of 80%. The precision level of estimate of 5% and the corresponding confidence interval of 95% were predetermined to assess association between patient’s related factors and adherence to dietary prescription. A 20% non-response was factored in based on the following formula: n* = *N*/(1 – q), where n* = adjusted sample size, *n* = sample size before adjusting, and *q* = the proportion expected for nonresponse. The sample size was proportionally allocated to the study sites, KNH and MTRH, based on the number of adults with CKD attending the renal care facilities. Participants who were on hemodialysis were identified from the health records. From each study site, participants were purposively sampled consecutively following the inclusion and exclusion criteria to achieve the desired sample size.

#### Study variables in quantitative approach

The dependent variable was “adherence to dietary prescriptions.” It was defined as “the extent to which dietary habits of adults with CKD corresponded with the recommended dietary intake for their disease condition, with the recommendations followed all the time in the last seven days prior to the survey.” During data collection, it was self-reported on a 5-point Likert scale, where 1 = adherence to dietary prescriptions, with the recommendations followed all the time; 2 = mild non-adherence, with the recommendations followed most of the time; 3 = moderate non-adherence, with the recommendations followed about half of the time; 4 = severe non-adherence with the recommendations followed very seldom; 5 = very severe non-adherence, with the recommendations not followed at any time in the past 7 days [31, 32]. A binary variable was computed from this 5-point Likert scale during data analysis as “1 = 1” coded as “adherence” for “recommendations followed all the time” and “2–5 = 0” coded as “non-adherence” for “recommendations not followed all the time.”

The independent variables were the participants’ characteristics of socio-demographic, access to dietary prescription information, health care systems, “perceptions” on dietary restrictions, and challenges to adherence to dietary prescription. The definitions of the variables on “perceptions” as used in this study are provided in Table 1.

#### Quantitative data collection

Data collected included the following variables “demographic characteristics, socio-economic information, clinical parameters of duration with CKD, co-morbidities and nutritional status, health care systems, access to fluid and dietary prescription information, perceptions to adherence to fluid and dietary prescription information, challenges to adherence to fluid and dietary prescription information (including level and type of difficulty experienced), and adherence to fluid and dietary prescription information.” The parameters on adherence were adapted from the validated end-stage renal disease-adherence questionnaire [31] and the dialysis diet and fluid non-adherence questionnaire [32]. Questions on perceptions about adherence to dietary prescriptions on the questionnaire were adapted from Sutton et al. [33]. Once each participant had completed responding to the questionnaire, the data were uploaded onto Google Sheets on “ODK Collect” software. Data were then downloaded as an “.xls” file for further cleaning and analysis.

#### Numeric data management and analysis

Analysis of the quantitative data was performed using STATA statistical software. Participants in adherence and non-adherence groups were summarized as counts (*n*) and percentages (%). Univariate and multivariate logistic regression models were constructed to assess the associations between potential contributing factors and adherence to dietary recommendations. In univariate models, factors with *P* <0.1 were considered statistically significant and were included in the multivariate logistic regression model as likely determinants of adherence. Factors with *P* < 0.05 were then considered independent determinants of adherence to dietary recommendations among adults with CKD.

### Qualitative approach

A qualitative descriptive approach, using in-depth interviews with open-ended questions, was used to triangulate the data obtained from the quantitative survey. Participants composed of CKD patients and their caregivers were purposively selected following the inclusion and exclusion criteria for the in-depth interviews. The inclusion and exclusion criteria for participants (who in this case were CKD patients) were the same as the one for those who responded to the questionnaire. However, CKD patients were excluded from the in-depth interviews if they had responded to the questionnaire. Caregivers were excluded if their patient had responded to the in-depth interviews. Caregivers were included in the study if they were household members, aged 18 years and above, accompanied the patient to the hospital, and provided voluntary informed consent.

An iterative approach involving repetitive interactions between the field research teams, principal investigator, and the person coding the data was adopted in data collection and sampling process. Initially, each interviewer selected a participant based on inclusion criteria and obtained consent from the participant to conduct the interview using the research study guidelines, note-taking and voice recording with a universal serial bus (USB) audio recorder. After the interview process, data were sent electronically to the field monitor who was monitoring the field data collection process. The field monitor then reviewed the data for quality control procedures to ascertain completeness and data quality. The PI of the project consolidated the feedback on the data quality as provided by the field monitor checking field operations and interview processes. Regular meetings, at least once every week, were held between the PI and the research team comprising of the field monitor, the coder, and the interviewers to ascertain the information emerging from the interviews and the main thematic coverage, emerging themes, socio-demographic, and co-morbidity representation of the data collected. The drive behind further selection of participants to be interviewed was determined by data saturation.

#### In-depth interview process for CKD patients and their caregivers

Data were collected using open-ended in-depth interview guides and voice recording on perceptions, beliefs, and factors influencing adherence to prescribed diets among CKD patients. The design of the questions for the interview guides was informed by three constructs: (i) the health belief model [34], (ii) the World Health Organization’s five dimensions of adherence to dietary prescriptions [17], and (iii) Sutton’s tool on patients’ perceptions of renal dietary advice [33]. The qualitative data collection covered access to dietary information, health care systems, perceptions and beliefs on dietary restriction, and challenges to adherence [17, 21, 34]. To enhance the depth of information collected, the interviewers varied the questions asked during the interview so as to stimulate participants to provide detailed information regarding their recommended diets.

#### Analysis of qualitative data

The qualitative data analysis was done using NVIV0 12 computer software. Thematic analysis was done based on existing themes that were decided a priori: perceptions and beliefs. Content analysis was applied to identify and categorize the emerging themes and sub-themes. Quasistatistics analysis was applied to count the number of times each item appeared regarding the participant characteristics. The health belief model was applied to describe contextual factors that influence participants’ decision-making regarding adherence to dietary recommendations [35].

## Results

### Study population

#### Study population in quantitative survey

The characteristics of the study population in the quantitative survey are presented in Table 2. There were 333 participants in this study, of which 59.8% were males. Most of the participants (66.4%) were living with their spouses. The mean (± SD) age was 46.7 (± 17.3) years. The majority of the participants (79.9%) had high blood pressure with BMI of 22.1 (± 3.8) kg/m^2^.

### Study population in qualitative survey

Table 3 shows the characteristics of participants in the qualitative survey. There were 92 participants in the qualitative survey (KNH, *n* = 63 and MTRH, *n* = 29). Among the participants, 52 were patients and 40 were family caregivers. The number of males was 42 while females were 50. These participants were aged 41 to 60 years old, and more than half of them were above 40 years of age (59/92). Majority of the participating patients (50/52) and caregivers (36/40) were aware of diet recommendations.

### Access to nutrition information and counseling services

Data on access to nutrition information and counseling from the quantitative survey is shown in Table 4. Almost all participants (92.8%) were aware of the dietary recommendations for patients with CKD. The main source of nutrition information was the nutritionist (90.3%) at the health facility. Other sources of information were doctors, nurses, media, and fellow patients that constituted 9.7% of the sample population. The study also observed that only 63.7% of participants reported that they frequently received nutrition counseling from the nutritionist, and slightly more than half (55.9%) of the participants indicated that nutrition counseling was affordable. The findings also indicate that just half of the participants were aware that the dialysis treatment package included nutrition counseling (50.5%) and that the National Hospital Insurance Fund (NHIF) medical cover pays for nutrition counseling (48.5%). It was however not clear from the qualitative interviews whether nutrition counseling was affordable or not although participants confirmed that at KNH, individualized nutrition counseling services cost an extra Kenya Shillings (KES) 300 ($3).

From the qualitative interviews (Table 5), it was evident that both the participating patients and caregivers were aware of the foods that the patient should eat as well as the methods of preparation. For example, they knew that the patients were allowed to eat beans soaked in water before cooking to remove potassium. Similarly, tomatoes were also leached in hot water to remove the top peel which is rich in potassium. Participants reported that they were allowed to eat boiled lean meat, chicken, or fish without fat. With regard to the vegetables, they were leached and the water used in cooking was discarded before frying the vegetables. According to both the participating patients and caregivers, they had been advised to avoid acidic fruits like oranges and lemons due to their high potassium content.

### Perceptions on dietary prescription

In the quantitative survey (Table 2), participants reported that they perceived the diet and fluid recommendations for adults with CKD on hemodialysis as generally important (diet = 83.1%; fluid = 77.7%). They were motivated to follow the recommendations because of the perceived health benefits for their health and kidneys (diet = 66.7%; fluid = 77.7%). However, majority of them (83.8%) felt that the diets were restrictive and only 16.2% of them considered the diets to be flexible enough to fit with other ways of eating. More than half of them had challenges (61.8%) in their attempts to follow the diet recommendations.

**Fig. 1:**
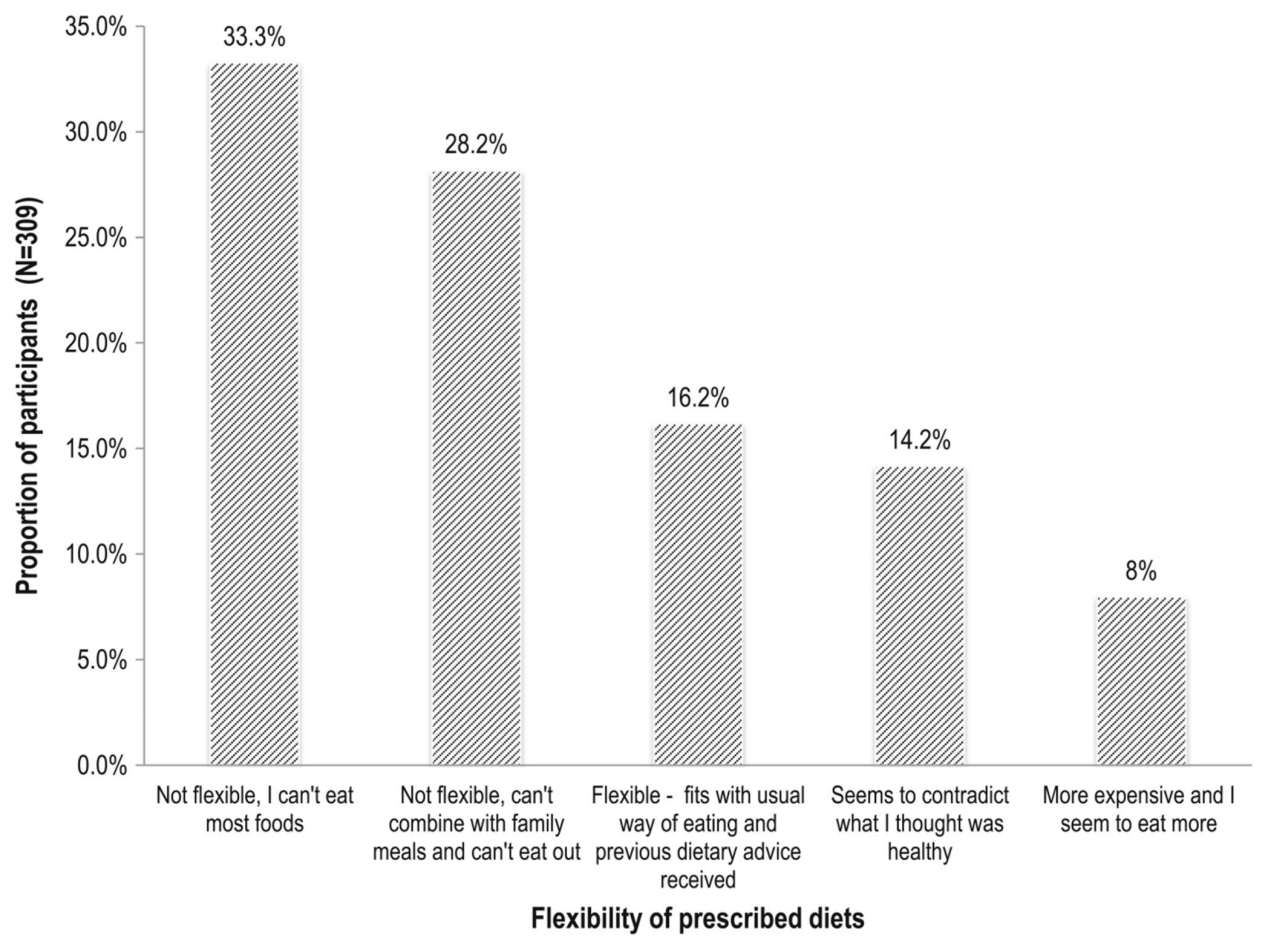
Flexibility of prescribed diets. This was assessed as the “flexibility of the prescribed diet to fit in with usual dietary habits” of the participants. It was assessed on a 9-point Likert scale [31, 33] of 1 = it fits in with my usual way of eating; 2 = it seems to contradict what I thought was healthy; 3 = it is difficult to combine with the rest of the family; 4 = it makes it difficult to eat out; 5 = it combines easily with other dietary advice I have been given; 6 = it is more expensive than my usual way of eating; 7 = I seem to have to eat more than I want; 8 = there are lots of foods I can no longer eat; 9 = I do not need to make any changes. During analysis, the responses for “1, 5, and 9” were combined to represent “flexible and fits with usual way of eating and previous dietary advice received”; “3 and 4” were combined to represent “not flexible, cannot combine with family meals, and cannot eat out.” Responses “6 and 7” were also combined to represent “more expensive and I seem to eat more”

Table 6 and Fig. 1 show that only 16.2% of study participants reported that the prescribed diets were not restrictive, but flexible and conformed to their usual ways of eating as well as previous dietary advice they had received. The rest of the participants, over 80%, found the diets to be restrictive because they could no longer eat most of the foods that they were used to (33.3%), could no longer share family meals together or eat away from home (28.2%), the diet seemed to contradict what they thought were healthy foods (14.2%), or that the foods were more expensive than their usual food items (8.0%).

The quantitative data also indicated that apart from the prescribed diets being restrictive, most participants, 61.8%, reported that they experienced challenges in following the diets. These challenges are highlighted in Table 7 where most participants felt that they were unable to avoid certain foods (39.0%) or fluid (67.5%). The prescribed food items were also not accessible due to either unavailability or cost (19.0%).

Qualitative findings also confirmed that the diets were challenging as most of the prescribed food items had to be purchased from the market and prepared separately because they did not fit with the family meals. The foods and cooking methods had to be different. This also made the prescribed diets stressful and expensive to prepare (Table 8).

### Adherence to diet prescription and fluid limitation

**Fig. 2:**
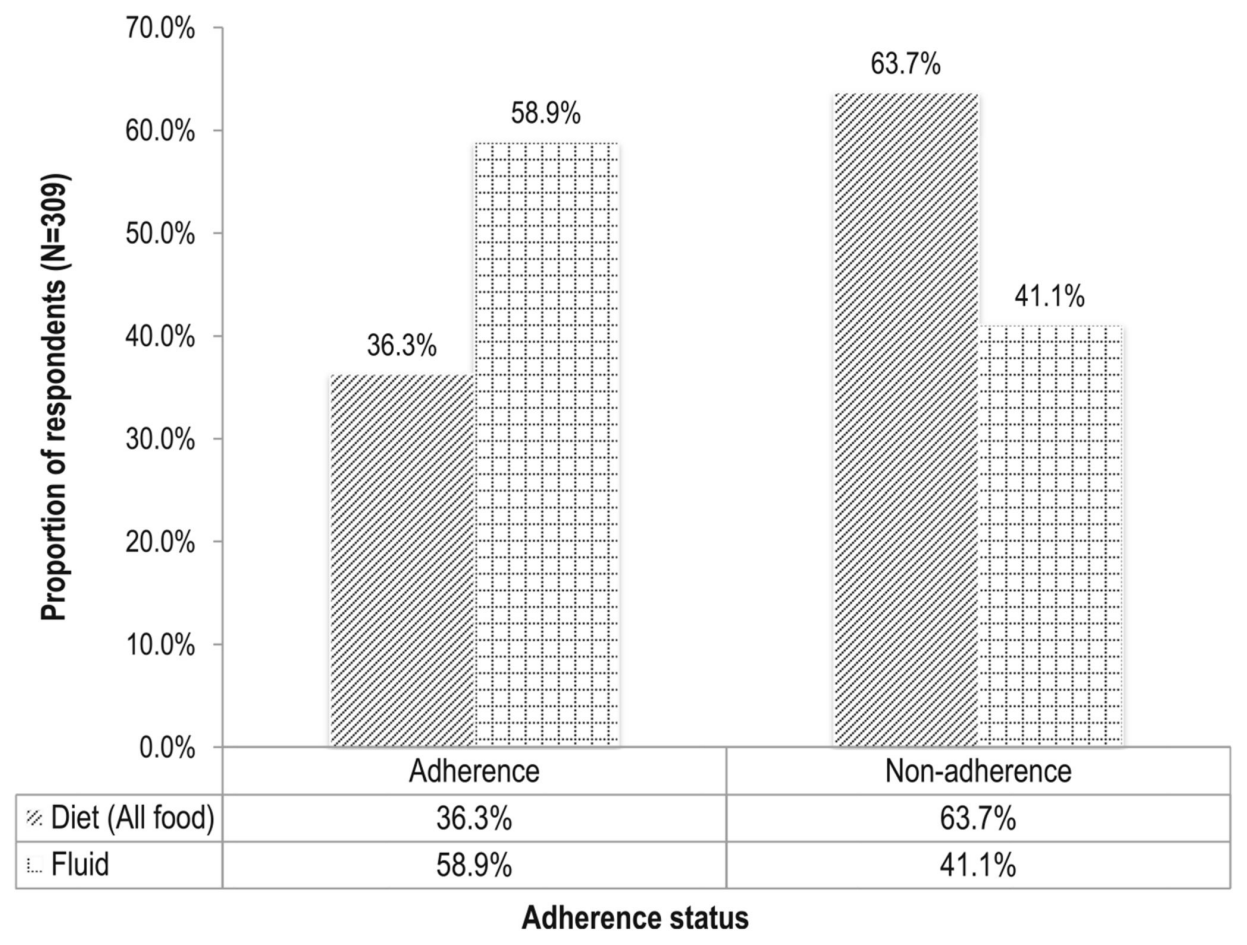
Adherence to diet prescription and fluid restriction. Adherence was self-reported on a 5-point Likert scale where 1 = adherence to dietary prescriptions, with the recommendations followed all the time; 2 = mild non-adherence, with the recommendations followed most of the time; 3 = moderate non-adherence, with the recommendations followed about half of the time; 4 = severe non-adherence with the recommendations followed very seldom; 5 = very severe non-adherence, with the recommendations not followed at any time in the past 7 days [7, 24]. A binary variable was computed from this 5-point Likert scale during data analysis as “1 = 1” coded as “adherence” for “recommendations followed all the time” and “2–5 = 0” coded as “non-adherence” for “recommendations not followed all the time.”

The proportion of participants, who reported that they adhered to their dietary prescriptions, having followed it all the time, was 36.3% (Figure 2). Non-adherence to diet was therefore 63.7%. For fluid limitation, adherence was 58.9% while non-adherence was 41.1%.

### Factors associated with adherence to dietary prescriptions

In the univariate analysis (Table 9), BMI, perceptions to limiting fluid intake, flexibility of the diets in fitting with other meals, difficulties in following the recommended diets, difficulties in limiting fluid intake, and adherence to fluid intake restrictions were significantly associated with adherence to dietary prescriptions.

Table 9 Univariate analysis of factors associated with adherence to dietary prescriptions.

### Multivariate analysis of factors associated with adherence to dietary prescriptions among adult patients with CKD on hemodialysis

In the multivariate regression analysis (Table 10), “flexibility in the diets to fit with other ways of eating” was significantly associated with adherence to dietary prescriptions (AOR 2.65, 95% CI 1.11–6.30, *P* 0.028). Furthermore, patients who experienced “difficulties in following diet recommendations” were significantly less likely to adhere to dietary prescriptions than those who did not experience difficulties (AOR 0.24, 95% CI 0.13–0.46, *P* < 001). The patients who reported that they “adhered to their fluid intake restrictions” as recommended were more likely to adhere to the diet recommendation than those who reported that they did not follow the recommendations for their fluid restriction (AOR 9.74, 95% CI 4.90–19.38, *P* < 0.001).

## Discussion

The study determined factors associated with adherence to renal dietary prescriptions among adult patients with CKD on hemodialysis. Overall, adherence to diet prescription was low among these patients who were all aware of the recommended foods for their health condition. Awareness of the foods and preparation methods was equally high among the family caregivers. Being aware of the right foods to consume as well as the right preparation methods for the CKD patients on hemodialysis did not translate into adherence to the dietary prescription. Similar findings have been reported by Beerendrakumar and colleagues [36]. Non-adherence to diet prescriptions is a critical shortcoming among CKD patients, and this pattern is apparent in studies reported from developed countries where more than half of adults on hemodialysis do not adhere to their diet prescriptions [10, 14, 16, 21, 37]. This raises concerns on the wellbeing and clinical stability of the CKD patients including the risk of co-morbidities. For the CKD patients on hemodialysis, limiting the intake of certain foods is important in order to reduce the accumulation of metabolic wastes in the blood as well as reduce the development of comorbidities such as hypertension and other health complications [11]. Although hypertension was the most common co-morbidity in our study, we did not find any statistically significant relationship between hypertension and adherence to dietary prescriptions. However, the qualitative findings indicated that severity of the kidney disease and associated co-morbidities was one of the consequences of consuming the restricted foods.

Factors that were independently associated with adherence to diet prescriptions in this study were *flexibility in the diets to fit with other ways of eating, difficulties in following diet recommendations*, and *adherence to fluid intake*. Those participants who perceived the prescribed diets to be flexible and matched with other ways of eating were more likely to adhere than those who considered the prescriptions to be restrictive (AOR 2.65, 95% CI 1.11–6.30, *P* 0.028). In this patient population, factors related to food types, food preparation methods, and social gatherings appeared to be major impediments among CKD patients abiding to diet prescriptions. The diets were equally restrictive for the caregivers who found it stressful, time consuming, and expensive to prepare. It is known that individuals with CKD often feel curtailed in their normal way of life as food restrictions make it difficult for them to modify lifestyle and fit in with their current clinical needs [38, 39]. From our findings, the proportion of participants who reported that the prescribed diets were restrictive was almost similar to the ones who reported non-adherence. These findings suggest that diets with fewer restrictions in terms of types of foods to consume and food preparation methods are more likely to be adhered to than the more restrictive ones.

Our study also observed that participants who experienced challenges in their attempts to adhere to the diet prescriptions were less likely to adhere than those who had no difficulties (AOR 0.24, 95% CI 0.13–0.46, *P* < 001). Among the reported difficulties were *inability to avoid certain types of restricted foods, inadequate information on the recommended diets*, and *cost and unavailability of the prescribed foods*. Limiting intake of certain foods such as red meat or dietary salt was a challenge for most CKD patients in our study. It was noted in this study that the recommended foods were unpalatable, for example, without salt additive. Although we did not assess adherence specific to dietary salt intake, our qualitative findings suggest that the patients did not adhere to dietary salt restrictions. Previous studies have reported non-adherence to dietary sodium intake among CKD patients [18, 40]. Intake of dietary salt above the recommended amount of 5 g per day may contribute to sodium retention due to poor excretion by the damaged kidneys [41] leading to fluid overload among CKD patients. Participants in our study were also likely to consume diets with high potassium levels since they found leaching green vegetables not only a challenge, but the leached vegetables were also unpalatable and contradicted what they thought were healthy ways of preparing food. The leaching of vegetables is however necessary to reduce the potassium content of food in order to avoid hyperkalemia which occurs in CKD patients due to reduced urinary potassium excretion [42]. Whereas the intention of leaching the vegetables is to reduce their potassium content, it also reduces the water-soluble vitamins C and B complex of these vegetables. No wonder the study participants claimed that the prescribed diets seemed to contradict what they knew to be healthy foods. However, with proper dietary counseling on the importance of leaching the vegetables and consumption of other foods rich in the water-soluble vitamins lost during leaching, both the patients and their caregivers can adhere to the prescribed diets. This implies that the prescribed diets should be rich in vitamin C and B complex to replace what is lost from the leached vegetables and to prevent development of anemia in these patients.

The inability to avoid certain restricted foods makes transition to the recommended diet a challenge, hence the observed low level of adherence to dietary prescriptions among adults on hemodialysis in our study. Our qualitative findings suggested that inability to limit intake of certain foods by the CKD patients was partly attributed to poor communication of nutrition information and subsequently poor understanding of the diet prescription messages. Existing literature shows that conflicting dietary advice from different health professionals is associated with poorer dietary adherence [13]. We also found that some of the prescribed diets were expensive and sometimes not available, thus limiting access to such foods that had to be acquired through purchase. It is likely that under such circumstances, when special diets are expensive or not available, people end up consuming the restricted food items that are accessible to them, hence non-adherence. Sullivan and colleagues [43] from the USA have reported a statistically significant variation in availability of unrestricted diet and renal diet food items in grocery stores. Poor access to the recommended food items for the CKD patients is likely to contribute to low consumption of such foods, hence non-adherence to the diet prescriptions.

The last independent predictor of adherence to diet prescriptions in our study was *adherence to fluid intake*. The participants who adhered to recommendations on limiting fluid intake were more likely to adhere to the prescribed diet (AOR 9.74, 95% CI 4.90-19.38, *P* < 0.001) than those who did not adhere to their fluid restrictions. These findings suggest that patients who adhere to their fluid restrictions have a better understanding of the potential health hazards of non-adherence to both diet and fluid restrictions than those who do not adhere [44]. Another possible explanation could be the effectiveness of counseling on fluid intake by the nephrology nurses who spend longer hours with the patients, encouraging them to limit their fluid intake [44, 45]. Furthermore, the counseling on fluid restriction accompanies the dietary sodium restriction. It is therefore possible that those participants who adhered to their fluid restriction were the same ones who reported adherence to diet prescription.

### Strengths and weaknesses of the study

To our knowledge, this was the third most recent study in Africa on adherence to diet prescription among patients with CKD on hemodialysis. In Kenya, this was the first one. The study was conducted at a time when the problem of CKD was on the rise in the country. Since nutrition is the most modifiable lifestyle factor in the management of CKD, it was important to understand whether and why the CKD patients adhere to their dietary prescription in the local context to guide on intervention strategies during nutrition counseling. The triangulation of quantitative findings with the qualitative findings in the mixed-methods approach in this study contributed to a comprehensive understanding of adherence to diet prescription in the study population. The participants’ voices, which were transcribed from the audiotapes, further emphasized the credibility of these findings. Our readers should however be aware of some limitations of this study. First, this study used subjective self-reported approach to obtain quantitative data on adherence. Hence, our findings may not be exactly the same as those where non-subjective approaches have been used. Furthermore, we did not investigate whether the adults with chronic kidney disease on hemodialysis adhered to a specific dietary guideline or nutrient. Our study focused on self-reporting on whether the overall dietary prescriptions were followed or not. Finally, our study sample included only those adults with CKD on hemodialysis; hence, this study may not be generalized to children or other adult patients with CKD who are not on hemodialysis.

## Conclusion

The awareness of renal diets is high among both patients and caregivers. However, adherence to the renal diet prescription is low. For patients with chronic kidney disease on hemodialysis, the diet prescriptions with fewer restrictive foods that require minimal extra effort and resources are more likely to be adhered to than the more restrictive ones that do not match with other ways of eating and food preparation methods. Patients who adhere to their fluid intake restrictions are also likely to follow their diet prescriptions. Based on these findings, we suggest that the prescribed diets should be guided by the patient’s usual dietary habits and assess levels of challenges in their food environment in using such diets. The diet-related messages should be integrated with fluid restriction messages to increase chances of adherence to the diet prescriptions. Further research should be conducted to understand the following: (1) a “contextually less restrictive diet” for patients with CKD on hemodialysis, (2) adherence to specific nutrient prescriptions in Kenya, and (3) the reasons why patients on hemodialysis are more likely to adhere to their fluid restriction than to dietary prescriptions.

## Figures and Tables

**Table 1:** Definitions of “perceptions on diet and fluid recommendations” variables

Variable	Definition
Perception [31] on whether renal diet is important	Data collected on a 5-point Likert scale of “1 = highly important; 2 = very important; 3 = moderately important; 4 = a little important; and 5 = not important” and a binary variable computed as “1 and 2 = 1” coded as “it is important” and “3–5 = 0” coded as “not so important”
Motivation [31] for following recommended diet	Data collected on a 7-point Likert scale of “1 = because I fully understand that my kidney condition requires to watch my diet; 2 = because watching my diet is important for my health; 3 = because a medical professional (doctor, nurse, or dietician) told me to do so; 4 = because I got sick after eating certain food that I was not supposed to eat; 5 = because I was hospitalized after eating certain food that I was not supposed to; 6 = I do not think watching my diet is important to me; 7 = other (specify): ____.” A binary variable computed as “1 and 2 = 1” coded as “perceived health benefits (important for health and kidneys)” and “3–7 = 0” coded as “other reasons (counseling, hospitalization)”
Perception [31] on whether limiting fluid intake is important	Data collected on a 5-point Likert scale of “1 = highly important, 2 = very important, 3 = moderately important, 4 = a little important, and 5 = not important” and a binary variable computed as “1 and 2 = 1” coded as “it is important” and “3–5 = 0” coded as “not so important”
Motivation for limiting fluid intake	Data collected on a 7-point Likert scale of “1 = because I fully understand that my kidney condition requires to watch my diet; 2 = because watching my diet is important for my health; 3 = because a medical professional (doctor, nurse, or dietician) told me to do so; 4 = because I got sick after eating certain food that I was not supposed to eat; 5 = because I was hospitalized after eating certain food that I was not supposed to; 6 = I do not think watching my diet is important to me; 7 = other (specify): ____.” A binary variable computed as “1 and 2 = 1” coded as “perceived health benefits (important for health and kidneys)” and “3–7 = 0” coded as “other reasons (counseling hospitalization)”
Perception to weight measurement	Data was collected on a 5-point Likert scale of “1 = highly important, 2 = very important, 3 = moderately important, 4 = a little important, and 5 = not important” and a Binary variable computed as “1 and 2 = 1” coded as “it is important” and “3–5 = 0” coded as “not so important”
Difficulty in watching diet intake	This was assessed as either “presence” and coded as “1” or “absence” and coded as “0” of difficulties in following diet recommendations.
Diet fits with other ways of eating [31, 33]	This was defined as the “flexibility of the diet to fit in with usual dietary habits” of the participants. This variable was assessed on a 9-point Likert scale of 1 = it fits in with my usual way of eating, 2 = it seems to contradict what I thought was healthy, 3 = it is difficult to combine with the rest of the family, 4 = it makes it difficult to eat out, 5 = it combines easily with other dietary advice I have been given, 6 = it is more expensive than my usual way of eating, 7 = I seem to have to eat more than I want, 8 = there are lots of foods I can no longer eat, 9 = I do not need to make any changes.” A Binary variable was computed as “1, 5, and 9 = 1,” coded as “yes diet is flexible” and “2, 3, 4, 6, 7, and 8 = 0,” coded as “diet is not flexible” and does not fit in with other eating habits.

**Table 2:** Distribution of study population by socio-demographic and clinical characteristics

Participants’ characteristics (*N* = 333)	Summary *n* (%)
Socio-demographics	
*Age in years (Mean ± SD)*	*46.7 (± 17.3)*
Study site	
KNH	201 (60.4)
MTRH	132 (39.6)
Gender	
Males	199 (59.8)
Females	134 (40.2)
Age groups	
Young adults (18 to 40 years)	140 (42.0)
Middle and older adults (41 and above years)	193 (57.9)
Marital status	
With spouse—married	221 (66.4)
No spouse—never married, widow/widowed	112 (33.6)
Education level	
Primary and below	135 (40.5)
Secondary and above	198 (59.5)
Current employment status	
Income earners (employed/self-employed/retired)	174 (52.3)
Non-income earners (not employed)	159 (47.7)
Family support available	
No	54 (16.2)
Yes	279 (83.8)
Peer support available	
No	99 (29.7)
Yes	234 (70.3)
Clinical parameters *BMI (kg/m^2^) mean (± SD)*	*22.1 (± 3.8)*
Diabetes	
No	249 (74.8)
Yes	84 (25.2)
Hypertension	
No	67 (20.1)
Yes	266 (79.9)
Both diabetes hypertension	
No	254 (76.3)
Yes	79 (23.7)
*Duration with CKD (median (IQR) in months*	*8 (3–22)*

The variables in italics are continuous data summarized as mean (±SD) or median (IQR). The rest of the variables are summarized as counts and percentages. *KNH* Kenyatta National Hospital, *MTRH* Moi Teaching and Referral Hospital, *SD* standard deviation, *BMI* body mass index, *IQR* interquartile range

**Table 3:** A summary of participants’ characteristics from qualitative survey

Participants’ characteristics (*N* = 92)		Patients (*N* = 52)	Caregivers (*N* = 40)	All (*N* = 92)
Study site	KNH	31	32	63
	MTRH	21	8	29
Gender	Male	29	13	42
	Female	23	27	50
Age	18–40 years	19	14	33
	41+ years	33	26	59
Education	Primary and below	19	14	33
	At least secondary	33	26	59
Had diabetes	Yes	9	N/A	9
Had hypertension	Yes	35	N/A	35
Diabetes and hypertension combined	Yes	11	N/A	11
Aware of diet recommendations	Yes	50	36	86

**Table 4:** Access to nutrition information and counseling services

Access to nutrition information variable (*N* = 309)^a^	*N* (%)
Aware of dietary recommendations (*N* = 333)	
No	24 (8.4%)
Yes	309 (92.8%)
Main source of nutrition information (*N* = 309)	
Not a nutritionist (doctors, nurses, friends, other patients, or media)	30 (9.7%)
Nutritionist	279 (90.3%)
Frequency of nutrition counseling (*N* = 309)	
Rarely or never	112 (36.3%)
Frequently	197 (63.7%)
Treatment includes nutrition counseling (*N* = 309)	
No	153 (49.5%)
Yes	156 (50.5%)
^b^NHIF insurance pays for nutrition counseling (*N* = 309)	
No	159 (51.5%)
Yes	150 (48.5%)
Nutrition counseling is affordable (*N* = 309)	
No	134 (43.4%)
Yes	175 (56.6%)

b *NHIF* National Hospital Insurance Fund

a The access to nutrition information variables were analyzed for only participants who reported that they were aware of dietary recommendations for patients with CKD on hemodialysis (309/333)

**Table 5:** Examples of quotes from the qualitative interview scripts that illustrate the awareness about dietary recommendations and appropriate cooking methods. The information in brackets () represent the respondent’s gender, whether patient or caregiver, age, and home county

“For food, I take rice, indigenous vegetables, beans, dengu (green grams). Beans and ndengu are soaked in water before cooking. I also eat very little meat like fish, chicken but the top layer is removed because it contains fat. The meat should be lean. The meat should be boiled then fried. I also take black beans which are prepared in the same manner. …. I don’t take maize because of the husk. For tomatoes you put them in hot water, then remove the top peel, then use it to cook…. To get rid of the potassium.” (Male Patient, 56, Nyandarua County) “Vegetables we boil for almost 15 minutes, we pour that water then we rinse. We use boiled water to rinse, then we pour the water and we put a small onion and small oil and the tomatoes. You have to remove the outer cover of the tomatoes.” (Female Caregiver, 45, Nyamira). “For fruits we give something like pawpaw and apples. We were told not to give high acid fruits like oranges and lemons because they are not good for the kidneys. They said such fruits increase the level of potassium in the body.” (Female Caregiver, 21, Nandi).

**Table 6:** Perceptions on diet and fluid recommendations

Perceptions^a^ on diet and fluid recommendations (*N* = 309)	*N* (%)
Perception on recommended diets	
It is Important	257 (83.2%)
Not so important	52 (16.8%)
Motivation for following recommended diet	
Perceived health benefits (important for health and kidneys)	206 (66.7%)
Other reasons (counseling hospitalization)	102 (33.3%)
Perception on limiting fluid intake	
It is important	240 (77.7%)
Not so important	69 (22.3%)
Motivation for limiting fluid intake	
Perceived health benefits	240 (77.7%)
Other reasons (counseling hospitalization)	69 (22.3%)
Perception on flexibility of diets, fit with other ways of eating	
Yes	50 (16.2%)
No	259 (83.8%)
Difficulty in watching diet recommendations	
No	118 (38.2%)
Yes	191 (61.8)

a The definitions of the variables in Table 5 as used in this study are found in Table 1

**Table 7:** Reported difficulties in prescribed diet and fluid restrictions by participants

Types of difficulties experienced	Diet, *N* = 205; *n* (%)	Fluid, *N* = 117; *n* (%)
Not willing to control what to eat or drink	27 (13.2)	1 (0.8)
Unable to avoid certain foods or fluid	80 (39.0)	79 (67.5)
Recommendation not understood	34 (16.6)	26 (22.2)
Recommended foods not available or expensive	39 (19.0)	–
Others (no appetite, monotony, no one to cook, not been advised, thirst)	25 (12.2)	11 (9.5)

**Table 8:** Examples of quotes from qualitative interview scripts that illustrate the restrictiveness of the prescribed diets. The information in brackets () represents the respondent’s gender, whether patient or caregiver, age, and home county

“So they make food separately for me, they don’t mix with that for other people because them they may want tomatoes but for me I want plain. I don’t want tomatoes; I want plain with very little oil that is what I take.” (Male Patient, 60, Bomet, Secondary, Retired). “You cannot cook for everyone else like that. So I have to have his own cooking pan, I cook his things and put them aside before I begin cooking for the others.” (Female Caregiver, 58 Nakuru, Unemployed). “It’s a bit stressful because you know now you’ll have to prepare two meals—different meals and that’s a budget above what we used to have. For example if we used to buy two tomatoes and cook for the whole family, you’d use the two tomatoes and an extra tomato and tomatoes used to go for KES 5.00 and now it’s KES 10.00. You see as life becomes more costly and then you’re given an extra burden, life becomes a bit difficult.” (Female, Caregiver, 21).

*KES* Kenya Shillings

**Table 9:** Univariate analysis of factors associated with adherence to dietary prescriptions

Variable (*N* = 309)	Unadjusted OR (95% CI)	*P* value
Socio-demographic factors		
Hospital		
MTRH	ref	
KNH	0.99 (0.62-1.58)	0.970
Gender		
Male	ref	
Female	0.88 (0.55-1.417)	0.611
Age in years	0.99 (0.97-1.00)	0.195
Age groups		
18–40 years	ref	
Above 40 years	0.86 (0.54-1.38)	0.547
Marital status		
No spouse	ref	
With spouse	0.70 (0.43-1.14)	0.156
Education level		
Primary and below	ref	
Secondary and above	0.94 (0.58-1.50)	0.799
Current employment status		
No income	ref	
Earning income	0.99 (0.62-1.58)	0.985
Family support available		
No	ref	
Yes	1.39 (0.72-2.68)	0.317
Peer support available		
No	ref	
Yes	1.31(0.78 - 2.19)	0.296
Clinical parameters		
Diabetes		
No	ref	
Yes	1.22 (0.72 - 2.07)	0.458
Hypertension		
No	ref	
Yes	1.17 (0.64-2.12)	0.601
Both diabetes and hypertension		
No	ref	
Yes	1.33 (0.78-2.26)	0.293
Duration with CKD		
Up to 6 months	ref	
More than 6 months	1.05 (0.66-1.69)	0.808
BMI (kg/m2)	1.06 (1.00-1.13)	0.030*
Access to nutrition counseling services		
Main source of nutrition information		
Not nutritionist	ref	
Nutritionist	0.71 (0.33-1.54)	0.397
Frequency of nutrition counseling		
Rarely	ref	
Frequently	1.10 (0.67-1.78)	0.695
Treatment package has nutrition counseling		
No	ref	
Yes	1.28 (0.80-2.04)	0.292
NHIF insurance cover counseling costs		
No	ref	
Yes	1.22 (0.77-1.95)	0.390
Nutrition counseling affordable		
No	ref	
Yes	1.54 (0.96-2.49)	0.071*
Perception to diet and fluid restriction		
Perception to diet restriction		
Not important	ref	
Important	0.89 (0.48 - 1.64)	0.716
Motivations for diet restriction		
Other reasons like hospitalization	ref	
Perceived health benefits	1.15 (0.70 - 1.90)	0.558
Perception to limiting fluid intake		
Not important	ref	
Important	1.90 (1.15 - 3.14)	0.012*
Motivations for limiting fluid intake		
Other reasons like hospitalization	ref	
Perceived health benefits	1.52 (0.85 - 2.72)	0.156
Perception to weight measurement		
Not important	ref	
Important	0.97 (0.51 - 1.83)	0.938
Flexible diets, fits with other ways of eating		
No	ref	
Yes	5.51 (2.84 - 10.68)	0.0001*
Difficulties following diet recommendations		
No	ref	
Yes	0.18 (0.10 - 0.29)	0.0001*
Difficulty limiting fluid intake		
No	ref	
Yes	0.59 (0.37 - 0.95)	0.032*
Adherence to fluid restriction		
Not adhered	ref	
Adhered	9.42 (5.03 - 17.63)	0.0000*

*OR* odds ratio, 95% *CI* 95% confidence interval, *Ref* reference, *BMI* body mass index

* Statistical significance at *P* value less than 0.1 and considered as likely determinants of adherence for inclusion in the multivariate model

**Table 10:** Multivariate analysis of factors associated with adherence to diet prescriptions among adult patients with CKD on hemodialysis

Factors associated with adherence (*n* = 309)	AOR (95% CI)	*P* value
Flexible diets, fits with other ways of eating		
No	Ref	
Yes	2.65 (1.11–6.30)	0.028**
Difficulties following diet recommendations		
No	Ref	
Yes	0.24 (0.13–0.46)	0.0000**
Adherence to fluid intake restriction		
Not adhered	Ref	
Adhered	9.74 (4.90–19.38)	0.0000**

*AOR* adjusted odds ratio, *95% CI* 95% confidence interval, *Ref* reference

** Statistically significant at *P* < 0.05

## Data Availability

Data is available upon request from the corresponding author at roseopiyo@uonbi.ac.ke, ropiyo@cartafrica.org

## Abbreviations

AOR: Adjusted odds ratio
BMI: Body mass index
CI: Confidence interval
CKD: Chronic kidney disease
ERC: Ethics and Research Committee
IREC: Institutional Research and Ethics Committee
KDIGO: Kidney Disease Improving Global Outcomes
KES: Kenya Shillings
KNH: Kenyatta National Hospital
MTRH: Moi Teaching and Referral Hospital
NHIF: National Hospital Insurance Fund
ODK: Open Data Kit
OR: Odds ratio
PI: Principal investigator
qual: Qualitative
QUAN: Quantitative
